# Autoimmune Hepatitis in a Patient With Cryoglobulinemic Vasculitis: A Rare Association

**DOI:** 10.7759/cureus.45905

**Published:** 2023-09-25

**Authors:** Arumugam Jeyapraniya, Shamila De Silva

**Affiliations:** 1 General Medicine, Colombo North Teaching Hospital, Ragama, LKA; 2 Medicine, University of Kelaniya, Ragama, LKA

**Keywords:** urticaria, vasculitis, cryoglobulinemia, hepatitis, autoimmune

## Abstract

When a patient with cryoglobulinemic vasculitis develops a concurrent liver disease, it is typically associated with hepatitis C. Here, we report the case of a patient with cryoglobulinemic vasculitis and autoimmune hepatitis. A 54-year-old previously healthy woman presented with chronic urticaria. A skin biopsy revealed leukocytoclastic vasculitis and elevated serum cryoglobulins, leading to a diagnosis of cryoglobulinemic vasculitis. She also had abnormal liver functions, high IgG levels, positive antinuclear antibodies, and anti-smooth muscle antibodies. Liver biopsy revealed interface hepatitis confirming the diagnosis of autoimmune hepatitis. This case represents the rare occurrence of autoimmune hepatitis in a patient with cryoglobulinemic vasculitis.

## Introduction

Cryoglobulinemia is a disease characterized by the presence of cryoglobulins in the serum [1]. Cryoglobulinemic vasculitis is characterized by the precipitation of cryoglobulins in small to medium-sized blood vessels, leading to end-organ damage [1]. Approximately 80% of cryoglobulinemic vasculitis cases are caused by hepatitis C virus (HCV), B-cell lymphoproliferative disorders, autoimmune diseases, and other infections [2]. Autoimmune hepatitis (AIH) is a rare inflammatory liver disease mediated by the immune system, distinguished by the presence of circulating autoantibodies, elevated IgG levels, and distinct histological characteristics [3]. The association of AIH and cryoglobulinemia is rarely described in the literature. We report an intriguing case in which a patient with cryoglobulinemic vasculitis presented with liver abnormalities secondary to AIH. Diagnosing this condition can be challenging in patients with cryoglobulinemic vasculitis, as liver damage is typically more associated with HCV than with other causes.

## Case presentation

The patient presented with chronic urticaria persisting for five months, generalized body weakness, arthralgia, loss of appetite, and a weight loss of approximately 5 kg over the course of one month. She also complained of vague abdominal pain in the right hypochondrial region but denied experiencing fever, ulcers, bilateral lower limb numbness, urinary symptoms, nausea, or vomiting. Furthermore, she did not report any symptoms indicative of anemia or hypothyroidism. The patient denied any features suggestive of chronic liver or kidney disease. She did not consume alcohol.

**Figure 1:**
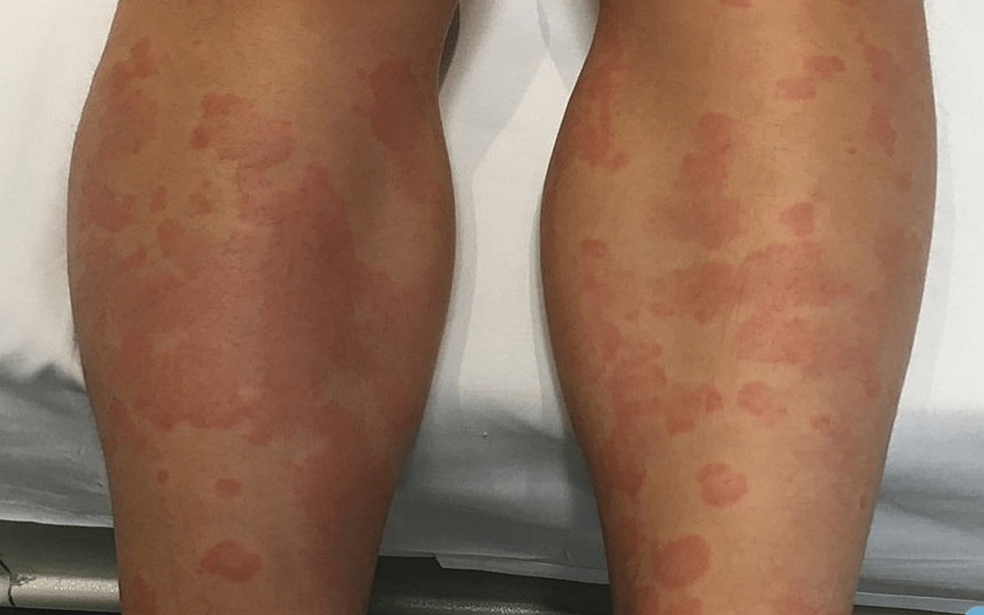
Chronic skin rash over the shin.

Upon examination, she was found to be afebrile, not pale, not icteric, without lymphadenopathy, and not dyspneic. A chronic urticarial rash was observed on her bilateral lower limbs (Figure 1). There were no features of connective tissue disorder or peripheral stigmata of chronic liver or kidney disease. Her pulse rate was 68 beats/minute with good volume, and her blood pressure was 130/80 mmHg. During the respiratory examination, a normal respiratory rate and clear lung fields were noted. Her abdomen was soft and non-tender, with mild hepatomegaly present but no splenomegaly. Neurologically, she exhibited normal findings.

The initial evaluation of chronic urticaria revealed normochromic normocytic anemia with a normal mean corpuscular volume. The patient also had altered liver function tests, including elevated levels of aspartate transaminase (AST) and alanine transaminase (ALT) (ALT > AST), with normal alkaline phosphatase, a high globulin level, mildly elevated gamma-glutamyl transferase, and normal C3 with low C4. However, renal function tests and thyroid-stimulating hormone were within normal limits, and the erythrocyte sedimentation rate (ESR) was 40 mm/hour. Upon further evaluation of chronic urticaria, we conducted tests for antinuclear antibodies and perinuclear antineutrophil cytoplasmic antibodies (P-ANCA). Both tests came back positive. Table 1 presents the laboratory findings.

**Table 1:** Investigation summary.

Investigations	Value	Reference
Hemoglobin	9.7 g/dL	13–16 g/dL
Mean corpuscular volume	87 fL	80–100 fL
Erythrocyte sedimentation rate	45 mm/hour	<30 mm/hour
Aspartate aminotransferase	234 U/L	0–35 U/L
Alanine aminotransferase	345 U/L	0–40 U/L
Total protein	6.7 g/dL	6–8.5 g/dL
Serum potassium	4.3 mmol/L	3.5–4.1 mmol/L
Serum sodium	134 mmol/L	137–145 mmol/L
Serum chloride	96 mmol/L	96–106 mmol/L
Serum calcium	2.2 mmol/L	2.15–2.57 mmol/L
Serum magnesium	0.9 mmol/L	0.8–1.2 mmol/L
Serum globulin	3.6 g/dL	2.5–3.5 g/dL
Total bilirubin	17.7 µmol/L	5–19 µmol/L
Direct bilirubin	12.5 µmol/L	0–5 µmol/L
Indirect bilirubin	5.2 µmol/L	0–5 µmol/L
Gamma-glutamyl transferase	256 U/L	5–40 U/L
Alkaline phosphatase	75 IU/L	44–170 IU/L
Serum creatinine	84 µmol/L	70–115 µmol/L
Creatinine phosphate kinase	200 U/L	<240 U/L
C3	118 mg/dL	75–175 mg/dL
C4	12 mg/dL	15–45 mg/dL
Thyroid-stimulating hormone	2.7 mIU/L	0.4–4 mIU/L
Ultrasound of the abdomen	Mild hepatomegaly	
Perinuclear antineutrophil cytoplasmic antibodies	Positive	
Hepatitis B surface antigen	Negative	
Hepatitis C antibodies	Negative	
C-reactive protein	2.3 mg/L	<5 mg/L
Urine full report	Albumin: Nil, Pus cells: 30–40, Red cells: 1–2	
Random blood sugar	162 mg/dL	
Rheumatoid factor	Negative	
IgG	251 g/L	6–16 g/L
Anti-mitochondrial Antibody	Negative	
Antinuclear antibodies	Positive	
Anti-smooth muscle antibody	Weakly positive	
DS-DNA	Negative	
Liver biopsy	Prominent interface and zone 1 lobular hepatitis. Hepatocyte necrosis in periportal area suggestive of Autoimmune hepatitis	
Serum cryoglobulin	Positive	
Skin biopsy and immune fluorescence	Leukocytoclastic vasculitis. Immunofluorescence testing showed deposits of IgM, IgG, and C3	
Hepatitis C RNA in serum and cryoprecipitate	Negative	

**Figure 2:**
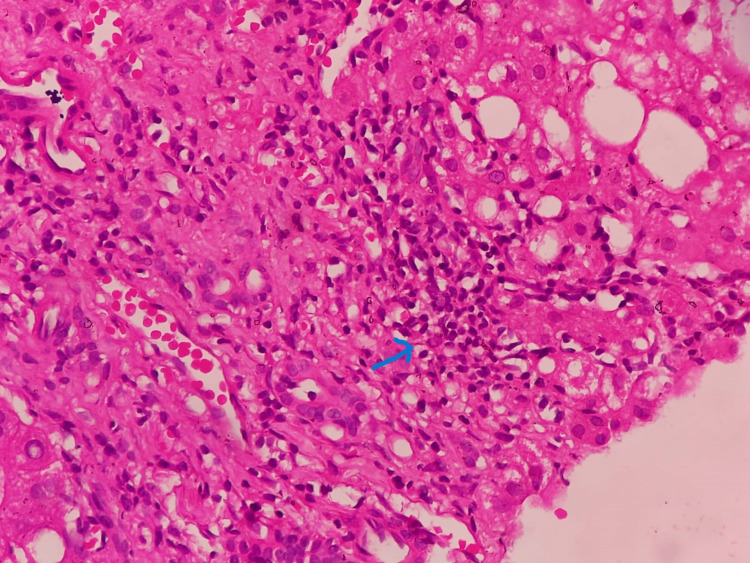
Liver histology showing interface hepatitis marked by blue arrow.

Subsequently, a skin biopsy was performed, revealing leukocytoclastic vasculitis. Immunofluorescence testing showed deposits of IgM, IgG, and C3. Serum cryoglobulins also tested positive, leading to the diagnosis of cryoglobulinemic vasculitis. Considering the abnormal liver function tests, further evaluation was conducted, revealing positive results for anti-smooth muscle antibodies, while antimitochondrial antibodies were negative. The patient had an elevated IgG level of 251 g/L (normal range = 6-16 g/L). Hepatitis B and C serology were negative, and hepatitis C RNA in serum and cryoprecipitate also tested negative. Therefore, a liver biopsy was performed, which demonstrated prominent interface and zone 1 lobular hepatitis, along with hepatocyte necrosis in the periportal area, suggestive of AIH (Figure 2).

She was started on prednisolone at a dose of 1 mg/kg (60 mg), which was gradually tapered off over three months. Azathioprine was initiated at a dose of 50 mg. Once prednisolone was discontinued, the azathioprine dose was increased to 100 mg/day and maintained at that level. She responded well to immunosuppressive agents, and her liver function and chronic urticaria symptoms improved. Follow-up appointments were scheduled at the gastroenterology clinic.

## Discussion

Cryoglobulinemia and HCV are commonly associated conditions, with HCV infection being a major trigger for the development of mixed cryoglobulinemia [1]. The majority of cases of mixed cryoglobulinemia, the most prevalent type, are indeed associated with HCV infection, estimated to affect up to 90% of patients with mixed cryoglobulinemia [2]. However, in our patient, both the hepatitis antibody test and HCV RNA test yielded negative results. Consequently, further investigation was warranted, leading us to identify AIH as the underlying cause for the altered liver functions in our patient. AIH is a rare immune-mediated inflammatory disease of the liver characterized by circulating autoantibodies, increased concentration of IgG, and distinctive histological features [3]. Extrahepatic involvement and associations with rheumatic diseases, such as Sjogren’s syndrome (SS), systemic sclerosis, systemic lupus erythematosus (SLE), and rheumatoid arthritis, are well-known. However, the coexistence of autoimmune liver disease (AILD) with small-vessel vasculitis in the same patients has only been occasionally reported [4]. Our patient presented with a rare combination of these two diseases. Our patient had a marginally elevated ESR due to AIH. In the case of other autoimmune diseases, such as SLE, we would typically expect higher values than this.

The triad of purpura, arthralgia, and weakness (Meltzer’s triad) is well-known in mixed cryoglobulinemia but is observed in only one-third of patients [5]. Interestingly, our patient did not fulfill this triad. The prevalence of cryoglobulinemia in primary SS is around 16% [6]. The second autoimmune disease strongly associated with cryoglobulinemia is SLE [7]. Anecdotal reports have also mentioned the presence of cryoglobulins in autoimmune diseases other than SLE or primary SS [8]. As our patient with cryoglobulinemic vasculitis was found to have AIH, it represents a rare presentation. Therefore, it serves as a reminder to physicians to consider a workup for AIH in a patient with cryoglobulinemic vasculitis if they present with altered liver function tests.

The association of cryoglobulinemia with other causes of chronic liver disease, such as hepatitis B and alcohol, is uncommon [9]. Trejo et al. evaluated 443 patients with cryoglobulinemia and found that 331 patients (75%) had infectious diseases, with the majority being hepatitis C. The authors did not identify any patients with AIH [10].

There are three other instances documented in the literature that fascinatingly illustrate the interplay between cryoglobulinemic vasculitis and AIH. Among them, an interesting report showed a 73-year-old gentleman presenting with palpable purpura on his lower limbs, accompanied by a surge in aminotransferases strikingly resembling the clinical presentation observed in our current case [10]. Equally compelling, Biecker et al. shed light on an extraordinary case in which the coexistence of celiac disease, known for its potential to incite autoimmune disorders, intertwined with AIH and cryoglobulinemic vasculitis [11]. Furthermore, Evans et al. fluently recorded an extraordinary encounter with AIH and cryoglobulinemia, wherein the vasculitis triggered renal involvement, initiating a cascade of glomerulonephritis and anuric acute kidney injury [11].

Single-nucleotide polymorphisms in CTLA-4 have been suggested as a genetic non-HLA-related risk factor in AILD [10]. This finding suggests that CTLA-4 alterations may be a common pathogenic pathway in the development of both AILD and systemic vasculitis. Finally, ANCA has been proposed as a possible further link [11]. Our patient also had positive P-ANCA. However, reduced complement levels, positive cryoglobulin in serum, and IgG, IgM, and C3 deposits on the immunofluorescence test led to the diagnosis of cryoglobulinemic vasculitis rather than ANCA vasculitis in our patient.

## Conclusions

This is an interesting case of cryoglobulinemic vasculitis with hepatic involvement, challenging the conventional association with HCV. Navigating the complex laboratory evaluation and liver biopsy findings revealed an enigmatic underlying culprit, AIH. This case report serves as a reminder to physicians that despite its rarity AIH should always be thought of when assessing patients with HCV-negative cryoglobulinemia.
